# Evaluation of the Effects of *Epicoccum nigrum* on the Olive Fungal Pathogens *Verticillium dahliae* and *Colletotrichum acutatum* by ^1^H NMR-Based Metabolic Profiling

**DOI:** 10.3390/jof11020129

**Published:** 2025-02-08

**Authors:** Federica Angilè, Mario Riolo, Santa Olga Cacciola, Francesco Paolo Fanizzi, Elena Santilli

**Affiliations:** 1 Department of Biological and Environmental Sciences and Technologies, University of Salento, Prov.le Lecce-Monteroni, 73100 Lecce, Italy; federica.angile@unisalento.it; 2 Department of Agriculture, Food and Environment, University of Catania, 95123 Catania, Italy; mario.riolo@unict.it (M.R.); olga.cacciola@unict.it (S.O.C.); 3 Council for Agricultural Research and Economics, Research Centre for Olive, Fruit and Citrus Crops (CREA-OFA), Rende, 87036 Cosenza, Italy

**Keywords:** NMR-based metabolomics, BCA, fungal interaction, *Epicoccum nigrum*, *Verticillium dahliae*, *Colletotrichum acutatum*, olive trees

## Abstract

Olive trees are a cornerstone of Mediterranean agriculture but face significant threats from diseases such as *Verticillium* wilt and olive anthracnose. These diseases, caused by *Verticillium dahliae* and *Colletotrichum* spp., respectively, result in significant economic losses and degrade olive oil quality. While traditional chemical treatments present environmental risk, sustainable alternatives such as biological control agents (BCAs) are gaining attention. *Epicoccum nigrum*, an antagonistic fungus, has shown potential as a BCA due to its production of antimicrobial secondary metabolites. This study aimed to observe whether *E. nigrum* has an antagonistic ability against *V. dahliae* and *C*. *acutatum,* and to elucidate the metabolic interactions between these fungi using NMR-based metabolomics. *E. nigrum* showed inhibitory effects on the growth of *C. acutatum* and *V. dahlia* of 44.97% and 38.73% respectively. Metabolomic profiling revealed distinct biochemical responses in *E. nigrum*, *V. dahliae*, and *C. acutatum* under mono- and dual-culture. Multivariate statistical analysis highlighted the metabolic shifts in mycelia and identified the primary metabolites, such as glutamine, 4-aminobutyrate, and phenylalanine that are involved in adaption for survival in stress conditions such as the presence of a competitor. The results could be important for a better understanding of the primary fungal metabolism, which is still poorly characterized. Further investigation is needed, but these results suggest that *E. nigrum* could serve as a BCA, offering a more sustainable approach to managing olive diseases.

## 1. Introduction

The olive (*Olea europaea* subsp. L. *europaea* var. *europaea*) has a rich history in the Near East, spreading westward into the Mediterranean basin, where it now thrives as a widely cultivated fruit tree [1]. Its ability to adapt to the dry and rural conditions of the region makes it not only an agricultural commodity but also a cultural and culinary icon that supports economies throughout the Mediterranean [2]. Globally, more than 11.6 million hectares of olive cultivation were reported according to data from the International Olive Oil Council [3].

Despite its agricultural importance, the olive tree is highly vulnerable to pests and diseases, two of the most severe being *Verticillium* wilt and olive anthracnose (OA).

The soil-borne, vascular-colonizing fungus *Verticillium dahliae* Kleb., particularly the highly virulent defoliating (D) pathotype, is the causal agent of *Verticillium* wilt of olive (VWO). *V. dahliae* can survive in the soil for many years, it has a wide host range, from annual plants to woody crops [4], and available fungicides are not effective [5]. The disease is considered one of the most threatening biotic constraints in many olive-growing countries [2]. This disease severely impacts olive trees by infecting their vascular system, leading to wilting, tree mortality, and reduced fruit yield. The disease not only causes substantial economic and cultural losses but also affects the quality of virgin olive oil [2,6].

Over the last two decades, *Verticillium* wilt has killed thousands of olive trees world-wide, with mortality exacerbated by factors such as the presence of susceptible cultivars, extensive irrigation, and the use of soils previously cultivated with susceptible annual hosts like cotton, tomato, or potato [2,7]. Valverde et al. (2023) report that disease incidence on olive trees varies significantly depending on irrigation practices, with rates of 9%, 17%, and 4% observed in non-irrigated orchards, compared to 21%, 40%, and 13% in irrigated orchards in Morocco, southern Spain, and Syria, respectively [7].

In addition to *Verticillium* wilt, olive anthracnose (OA) poses a major phytopathological concern, especially in the Mediterranean region. OA, predominantly caused by *Colletotrichum acutatum* and *Colletotrichum nymphaeae*, has a significant impact on olive cultivation globally [8,9]. Around 18 species of *Colletotrichum*, predominantly within the *C. acutatum*, *C. gloeosporioides*, and *C. boninense* species complexes, have been identified as causing olive anthracnose worldwide [8,9]. Alongside Mediterranean olive fruit fly and olive leaf spot, OA is a major concern for olive crops in the Mediterranean region [10,11]. OA leads to substantial deterioration in oil quality, influenced by the proportion of infected fruits, the specific *Colletotrichum acutatum* strains, and the olive cultivar [12,13,14]. In southern Italy, where conditions—such as warm, rainy, and humid climates during fruit ripening—favor disease development, OA outbreaks are endemic and severe [9,15,16]. Globally, *Colletotrichum acutatum* and *Colletotrichum nymphaeae* are the predominant species associated with OA outbreaks, particularly in the Mediterranean region. They are known for their aggressiveness and role in causing significant disease epidemics [8,9,11,17,18,19,20].

Olive anthracnose is a major disease of olive in Portugal, competing with *Bactrocera oleae* (Gmelin). With an average severity of 14%, OA causes 30–50% crop losses in Portugal but only 2.6% in Spain [13]. Anthracnose has been reported on all continents, with economically significant impacts in certain areas [9]. Expanding olive cultivation into new regions complicates predictions about OA incidence due to novel host–pathogen interactions [9,21].

Conventional management strategies for these diseases have largely relied on chemical treatments and the development of resistant cultivars. Presently, the management of olive diseases caused by fungi sensu latu, including fruit rots, primarily depends on pre-harvest treatments with copper and synthetic fungicides. However, European Directive 2009/128/EC, which aims to substantially reduce pesticide use in agriculture, has fostered the search for alternative, environmentally friendly, and toxicologically safer strategies. These alternatives include the use of generally recognized as safe (GRAS) natural substances and microorganisms or their metabolites that can stimulate natural plant defense mechanisms [22]. Consequently, there has been a growing interest in sustainable and environmentally friendly agricultural practices, particularly the use of biological control agents (BCAs) [23,24,25].

In recent years, there has been a growing interest in exploring natural and sustainable alternatives for managing plant diseases. One promising approach is the use of antagonistic fungi, or their secondary metabolites, as biocontrol agents [26,27,28,29,30]. Among the promising biological control agents (BCAs), *Epicoccum* spp., particularly *Epicoccum nigrum*, have gained recognition for their antagonistic properties against several fungal pathogens. These fungi produce a wide range of secondary metabolites with antimicrobial properties, such as β-carotene, γ-carotene, rhodoxanthin, torularhodin, epicocconone, and antibiotics like epicorazine A and B, and flavipin [31]. *E. nigrum* has shown efficacy in inhibiting the growth of several fungal pathogens, including *Fusarium* spp., *Botrytis cinerea*, *Rhizoctonia solani, Pythium* spp., and *Colletotrichum falcatum* [31,32,33,34,35]. Its potential extends beyond agriculture into pharmaceuticals and other industrial applications, such as biotransformation of pharmaceuticals, biosynthesis of nanoparticles, degradation of biogenic amines in wine, and lipid synthesis for biodiesel production [33,36,37,38].

To address these challenges, biological control agents are being extensively researched for their potential to manage plant diseases sustainably. Recent studies have highlighted the effectiveness of various BCAs, such as *Trichoderma* spp., *Bacillus* spp., and non-pathogenic *Fusarium oxysporum*, in controlling *Verticillium* wilt. These BCAs exert their effects through mechanisms such as antibiosis, competition, and induction of plant defense responses [2,22,23]. Similarly, other biocontrol agents are being explored for their ability to manage olive anthracnose by inhibiting the growth of *Colletotrichum* species [39].

Metabolomics, an advanced omics technology following genomics, proteomics, and transcriptomics, offers a powerful tool to study the biochemical interactions between beneficial and pathogenic fungi. In recent years, metabolomics has largely been used in several research fields regarding fungi, such as the identification and classification of pathogens, metabolic pathways of fungi, plant–fungal interaction and the study of fungal natural products (secondary metabolites) [40,41,42]. Furthermore, metabolomics allows the analysis and identification of hundreds of metabolites in a single experiment, exploiting advances in analytical techniques, enhanced instrumentation and bioinformatics tools [43]. Avant-garde instrumental approaches for metabolomics studies include Liquid Chromatography-Mass Spectrometry (LC-MS), Gas Chromatography-Mass Spectrometry (GC-MS) and Nuclear Magnetic Resonance spectroscopy (NMR) [44]. Recently, NMR has been widely employed in metabolomics studies, being a non-destructive analytical technique [41,44,45]. Moreover, NMR allows identification of molecular components of biological complex matrices, assigning up to 20 and 100 metabolites in vivo and in vitro, respectively. The low sensitivity of NMR, when compared with MS, is overcome by new pulse sequences for observing hetero- or homo-nuclear correlation [41,44,45]. Furthermore, NMR coupled with Multivariate Statistical Analysis (MVA) provides quantitative insights into the dynamic metabolic responses of living systems to environmental stimuli or genetic modifications [40,45]. In the context of fungal interactions, metabolomics enables the comprehensive profiling of metabolites involved in pathogenicity and biocontrol mechanisms [46,47]. However, the molecular mechanisms and metabolic interactions underlying its biocontrol efficacy remain underexplored. The aims of the study were the following: (i) to assess the in vitro antagonistic ability of *Epicoccum nigrum* against *Colletotrichum acutatum* and *Verticillium dahlia*; (ii) to analyze and compare the metabolic profiles of *Epicoccum nigrum*, *Verticillium dahliae* and *Colletotrichum acutatum* in mono- and dual-culture conditions using NMR spectroscopy; (iii) to interpret the metabolic data by MVA to understand the biochemical responses and interactions among the fungal isolates in different culture conditions.

## 2. Materials and Methods

### 2.1. Fungal Isolates

This study includes isolates of three different fungal genera, *Colletotrichum acutatum* (C9D2C), *Verticillium dahliae* (ER 1357) and *Epicoccum nigrum* (RD4C). *Colletotrichum acutatum* isolate was identified in previous studies [9]. *Epicoccum nigrum* (RD4C) and *Verticillium dahlia* (ER 1357) were identified molecularly in this study. Fungal isolates were sourced from the Council for Agricultural Research and Economics, Research Centre for Olive, Fruit and Citrus Crops (CREA-OFA, Rende (CS), Italy), the Research Centre for Plant Protection and Certification (CREA-DC, Roma, Italy) and the Molecular Plant Pathology Laboratory of the Department of Agriculture, Food and Environment, University of Catania (Italy). All fungal isolates were obtained from olive trees. Specifically, *Epicoccum nigrum* was isolated from olive drupes, *Colletotrichum acutatum* from symptomatic drupes [9] and *Verticillium dahliae* from soil collected from a symptomatic olive tree.

All isolates were cultured on Potato Dextrose Agar (PDA) at 24 ± 1 °C in the dark for 15 days until their use for morphological and molecular characterization in in vitro tests.

### 2.2. Morphological Identification of Epicoccum nigrum and Verticillium dahliae

The isolates *Verticillium* ER 1357 and *Epicoccum* RD4C were characterized morphologically based on colony and microscopic features. Both isolates were grown on PDA in Petri plates at 25 °C in the dark for 14 days.

For *Verticillium* ER 1357, morphological identification focused on the analysis of conidia and microsclerotia. Microsclerotia were examined under a light microscope, while conidia were obtained by preparing spore suspensions in sterile distilled water (SDW).

For *Epicoccum* RD4C, observations included colony characteristics such as size, color, and hyphal morphology. The dimensions and morphological features of mature conidia were measured at ×400 magnification to evaluate variability in size and shape, following the methodology described by Yu et al. (2016) [48] for *Verticillium dahliae*.

### 2.3. Molecular Identification of Epicoccum nigrum and Verticillium dahliae

RD4C and ER 1357 isolates, which had not been previously identified, were grown on PDA for 15 days at 24 ± 1 °C. Then, the mycelium of the isolates was harvested with a sterile scalpel, and approximately 1 g (fresh weight) of lyophilized mycelium was ground to fine powder in liquid nitrogen. The genomic DNA was extracted following the manufacturer’s protocol and using a PowerPlant Pro DNA isolation Kit (MO BIO Laboratories, Inc., Hilden, Germany). The DNA was preserved at −20 °C. The ITS1–5.8S–ITS2 region was amplified with primers ITS1 and ITS4 [49] and sequenced to confirm the isolate species. PCR amplifications were performed on a GeneAmp PCR System 9700 (Applied Biosystems, Foster City, CA, USA). All PCRs were carried out using *Taq* DNA polymerase recombinant (Invitrogen, Carlsbad, CA, USA) in a total volume of 25 μL containing PCR buffer (1×), dNTP mix (0.2 mM), MgCl2 (1.5 mM), forward and reverse primers (0.5 μM each), *Taq* DNA polymerase (1 U) and 1 μL of genomic DNA. Reaction conditions were 94 °C for 3 min; followed by 35 cycles of 94 °C for 30 s, 58 °C for 30 s, and 72 °C for 30 s; followed by an additional 10 min extension at 72 °C. Amplified products were analyzed by agarose gel electrophoresis and single bands of the expected size were purified with the QIAquick PCR Purification Kit (Qiagen, Duesseldorf, Germany) and sequenced with both forward and reverse primers by Macrogen Europe (Amsterdam, The Netherlands). The ChromasPro v. 1.5 software was used to evaluate the reliability of sequences and to create consensus sequences.

For RD4C, the β-tubulin (Tub2) gene was also analyzed. Amplifications of Tub2 were performed using primers Btub2Fd/Btub4Rd [50]. PCR conditions for Tub2 included an initial denaturation step at 94 °C for 2 min, followed by 35 cycles of 94 °C for 30 s, 54 °C for 30 s, and 72 °C for 30 s, with a final extension at 72 °C for 2 min. The amplified Tub2 fragments were processed as described for ITS, including purification and sequencing.

The ITS and Tub2 sequences obtained in the present study were deposited in GenBank. The reference sequences of *Epicoccum* spp. and *Verticillium* spp. used to construct the multilocus phylogenetic tree are shown in Table 1. The phylogenetic tree for *Epicoccum* was constructed using a multilocus approach, while for *Verticillium* only the ITS region was used. MUSCLE was used for sequence alignment, and phylogenetic trees were generated with MEGA11.

### 2.4. In Vitro Antagonistic Ability

The selected strain of *E. nigrum* (RD4C) was screened for its ability to inhibit the mycelial growth (%) of *C. acutatum* (C9D2C) and *V. dahliae* (ER 1357) strains by in vitro dual- culture assays [22]. The percentage inhibition of growth was calculated using the following formula:

$$Inhibition\;of\;growth\;\left(\%\right)=\left(\;\frac{X-Y}{X}\right)\times 100$$

where *X* is the radial growth of pathogen alone without antagonist (control) and *Y* is the radial growth of pathogen along with antagonist.

The dual-culture test was carried out in 9 cm Petri dishes with 15 mL of PDA by placing 5 mm diameter agar plugs of each pathogen and antagonist taken from the margin of 1-week-old colonies grown on PDA. The dual-cultures were set up by placing pathogen and antagonist 4 cm apart on PDA plates. Single cultures of each pathogen were used as control. Plates were incubated at 24 ± 1 °C in the dark, and radial mycelial growth was measured when *C. acutatum* (C9D2C) and *V. dahliae* (ER 1357) mycelium of control cultures covered the whole Petri dishes (namely, 10 days for *C. acutatum* and 15 days for *V. dahliae* after incubation). Overall, the experimental set-up consisted of the following four treatments (including controls) made of 10 replicates each: (i) *C. acutatum* isolate C9D2C, (ii) *V. dahliae* isolate ER 1357, (iii) *C. acutatum* isolate C9D2C + *E. nigrum* isolate RD4C, and (iv) *V. dahliae* isolate ER 1357 + *E. nigrum* isolate RD4C.

### 2.5. Sample Preparation for NMR Analysis

Mycelium extracts, aqueous and lipid, were obtained according to a modified Bligh and Dyer two-step method, a standard procedure for metabolomics profiling of cellular substrates [54]. In particular, for each culture test (mono and dual-culture assay) two biological replicates were carried out. For each sample, ~100 mg of lyophilized material, in three technical replicates, were added to methanol (400 μL), deionized water (285 μL) and chloroform (400 μL). The solution was mixed and placed on ice for 10 min and then was centrifuged for 20 min at 10,000 rpm. The two phases obtained, aqueous and lipid, were separated and dried by a SpeedVac vacuum concentrator. Successively, aqueous extracts were dissolved in 500 μL K_2_HPO_4_ buffer 0.1 M (pH 7.4) in D_2_O containing 0.01% of salt of 3-trimethylsilylpropionic acid-d4 (TSP-d4) as internal reference (δ = 0.00). Lipid extracts were dissolved in 600 μL CDCl_3_/CD_3_OD (2:1) containing 0.03% *v*/*v* trimethylsilane as internal standard (TMS, δ = 0.00). Aqueous and lipid extracts were then transferred into 5 mm NMR tubes and analyzed.

### 2.6. NMR Measurement

The ^1^H NMR spectra, ZGCPPR and ZG for aqueous and lipid fraction, respectively, were recorded using a Bruker Avance III NMR spectrometer (Bruker, Ettlingen, Germany) operating at 600.13 MHz for ^1^H observation, equipped with a TCI cryoprobe, incorporating a z-axis gradient coil and automatic tuning-matching. Experiment was acquired in automation mode after loading individual samples on a Bruker Automatic Sample Charger, interfaced with the software IconNMR Version 5 (Bruker), at 300 K. In detail, for mycelium aqueous extracts, 1D sequence with pre-saturation and composite pulse for selection (zgcppr Bruker standard pulse sequence) was acquired, with 128 transients, 16 dummy scans, 5 s relaxation delay, size of Free Induction Decay (FID) of 64 K data point, a spectral width of 20.0276 ppm and acquisition time of 2.73 s. For each lipid extract, a standard 1D ^1^H one-dimensional spectrum was acquired by performing the following experimental condition: 64 scans, 16 dummy scans, 64 K time domain, spectral width of 20.0276 ppm, 2 s delay, p1 8 μs and 2.73 s acquisition time. After acquisition, the resulting FIDs were multiplied by an exponential weighting function corresponding to a line broadening of 0.3 Hz before Fourier transformation, automated phasing and baseline correction.

For both extracts, the metabolites were assigned on the basis of 2D NMR spectral analysis (2D ^1^HJres, ^1^H COSY, ^1^H-^13^C HSQC, and HMBC) and by comparison with published data [54,55,56,57]. The metabolite assignments were verified using the Human Metabolome Data Base (HMDB).

### 2.7. Data Processing and Multivariate Statistical Analysis

In order to reduce the data dimensionality, the bucketing process and subsequent normalization on the total area (sum of each resonance intensity) within each bucket were calculated. In particular, all the spectra were divided into buckets with uniform spectral width (0.04 ppm for both extracts) by using AMIX 3.9.15 (Analysis of Mixture, Bruker Biospin GmbH, Rheinstetten, Germany) software. For aqueous extracts, the region 10.00–0.5 ppm was analyzed, with the exclusions of 4.98–4.74 ppm and 3.70–3.30 ppm containing the residual peaks from the suppressed water and methanol resonances, respectively. Analogously, for lipid spectra, the range 5.80–0.5 ppm was considered, excluding the methanol resonance region 4.46–4.26 ppm. For both extracts, Multivariate Statistical Analyses were performed, using as input variables the Pareto scaled NMR bucketed data [54]. A principal component analysis (PCA) was preliminarily performed on the whole dataset, and orthogonal partial least-squares discriminant analysis (OPLS-DA) were used to more deeply examine differences among samples and to identify the metabolites that contribute to their discrimination. Multivariate data analysis was performed using SIMCA-14 software (Sartorius Stedim Biothech, Umeå, Sweden). Commonly, PCA is applied to obtain and demonstrate the systematic variation in matrix X consisting of rows (the considered observation) and columns (the variables) of the buckets obtained from NMR spectra. The OPLS-DA analysis allows filtering out of variations that are not directly connected to the response, and produces models of clearer interpretation, focusing the predictive information in one component [54,58]. The PCA and OPLS-DA quality model is defined by R^2^ and Q^2^ parameters. In detail, R^2^ is a cross-validation parameter defined as the portion of data variance explained by the models, and indicates the goodness of fit. On the other hand, the Q^2^ parameter is the portion of variance in the data predictable by the model [54,59]. The minimal number of components required can be easily defined since R^2^ (cum) and Q^2^ (cum) parameters display completely divergent behavior as the model’s complexity increases [59,60]. The results were reported by the optimal bi-dimensional scores plots and relative loading plots; the latter were used to identify the metabolites responsible for separation among sample groups [54,58].

## 3. Results

### 3.1. Morphological and Molecular Identification

The morphological characteristics of *Verticillium dahliae* ER 1357 were consistent with typical features of the species. On PDA, the colony initially appeared creamy white, then darkened and became compact and appressed to the agar. The colony produced abundant hyaline conidia, which were cylindrical to oval, measuring approximately 4.4 ± 1.23 μm in length, with a maximum length of 6.0 μm. Conidiophores were hyaline, verticillately branched, and contained two to four phialides per node. Microsclerotia were dark brown to black, spherical, and compact, measuring 20–100 μm in diameter, with aggregates of microsclerotia reaching up to 200 μm in diameter. These features align with the description of *V. dahliae* provided in the study by Inderbitzin et al. (2011) [52].

In the case of *Epicoccum nigrum* RD4C, the colony on PDA was initially white with cottony mycelium, which later turned reddish-orange after 14 days of incubation. The reverse of the colony also produced an orange pigment. The mycelium spread across the entire surface of the plate, covering an area of approximately 633 mm^2^. The colony produced numerous conidia, which were globular to subglobose or pyriform in shape, with some conidia being multicellular and resembling a football shape. The conidia measured 14.5 to 26.4 μm in length and 15.9 to 28.4 μm in width. Immature conidia often had a brown stalk cell. These morphological features are consistent with *E. nigrum* [61]. The morphological characteristics of both fungi are summarized in Table 2.

**Figure 1:**
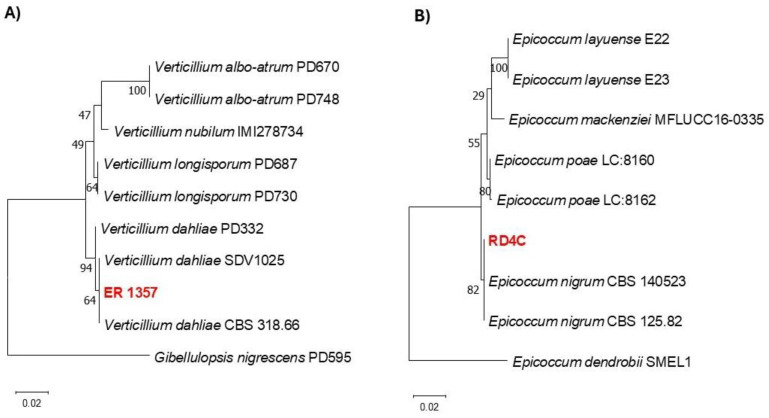
Phylogenetic trees of *Verticillium dahliae* (**A**) and *Epicoccum nigrum* (**B**) based on the internal transcribed spacer (ITS) and, for *E. nigrum*, also on the β-tubulin (Tub2) gene. In panel (**A**), the phylogenetic tree for *V. dahliae* was constructed using only ITS sequences, with *Gibellulopsis nigrescens* as the outgroup and a log likelihood of −1029.50. In panel (**B**), the multilocus phylogenetic tree of *E. nigrum* was developed using both ITS and Tub2 sequences, with *Epicoccum dendrobii* as the outgroup and a log likelihood of −1560.69. Maximum likelihood estimation was used to infer the trees, and the Tamura–Nei model was applied.

Molecular analyses were conducted on the RD4C and ER 1357 isolates. Phylogenetic analysis based on the ITS sequence grouped *Verticillium dahliae* ER 1357 with isolates SDV 1025 and CBS 318.66 (Figure 1A). Similarly, the multilocus phylogenetic analysis of *Epicoccum nigrum* RD4C, based on both ITS and Tub2 sequences, clustered it with the reference isolates CBS 140523 and CBS 125.82 (Figure 1B). The GenBank accession numbers for the ITS sequences of RD4C and ER 1357 are PP413698 and PP809450, respectively.

Additionally, the Tub2 gene sequence of *Epicoccum nigrum* RD4C was deposited in GenBank under Accession No. PP420949.

### 3.2. In Vitro Antagonistic Ability

**Figure 2:**
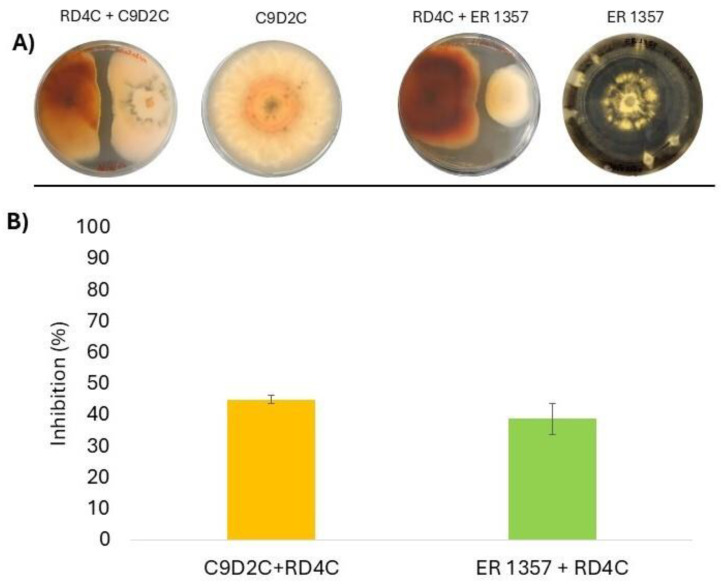
(**A**) Petri dish cultures: C9D2C + RD4C, dual-culture of *C. acutatum* and *E nigrum*; RD4C, mono-culture of *E nigrum;* ER 1357 + RD4C, dual-culture of *V. dahliae* and *E nigrum;* ER 1357, mono-culture of *V. dahliae.* (**B**) Percentage of mycelial growth inhibition of *Colletotrichum acutatum* (C9D2C) and *Verticillium dahliae* (ER 1357) observed in the dual-culture test against *Epicoccum nigrum* (RD4C). Inhibition values were calculated after 10 days for *C. acutatum* and 15 days for *V. dahliae* following incubation.

In dual-culture assays, *E. nigrum* (RD4C) inhibited the growth of *C. acutatum* by 44.97 ± 1.37% and *V. dahliae* by 38.73 ± 4.77% (Figure 2A), showing significant antagonistic potential (Figure 2B).

### 3.3. NMR Profiles Obtained from E. nigrum, V. dahliae, C. acutatum

**Figure 3:**
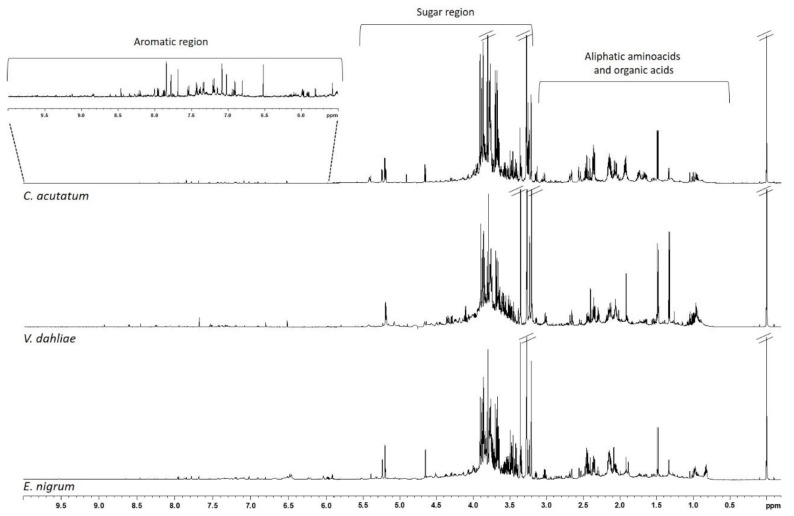
Typical ^1^H-NMR ZGCPPR spectra of aqueous extracts of three pathogens: *E. nigrum* (**bottom**), *V. dahliae* (**middle**) and *C. acutatum* (**top**); a representative expansion of aromatic region is also shown.

NMR-based metabolomics was applied to explore metabolic profiles of mycelia. Both aqueous and lipid extracts were considered for *E. nigrum*, *V. dahliae* and *C. acutatum* (mono- and dual-culture assay) obtaining a specific profile. Typical ^1^H-NMR ZGCPPR (600 MHz) spectra of aqueous extracts obtained from mycelia samples are reported in Figure 3. The aqueous fraction was dominated, in the high and middle region (0.00–5.60 ppm), by signals ascribable to amino acids, organic acids (succinate, lactate) and sugars (mainly α and β glucose, mannitol and trehalose). The low-field aromatic region (6–10 ppm) showed signals assigned to phenolic compounds such as aromatic amino acids (phenylalanine and tyrosine), and alkaloids (trigonelline) (Table 3). Furthermore, NMR spectra obtained from *E. nigrum* extracts showed a typical signal of propanol (δ = 0.83). Moreover, the ^1^H NMR spectra of aqueous extracts of *V. dahliae* were characterized by signals ascribed to nicotinate at 8.94, 8.61, 8.25 and 7.53 ppm, that were absent in *E. nigrum* and *C. acutatum* extracts. A malonate signal at 3.12 ppm was found only in extracts of *C. acutatum*.

**Figure 4:**
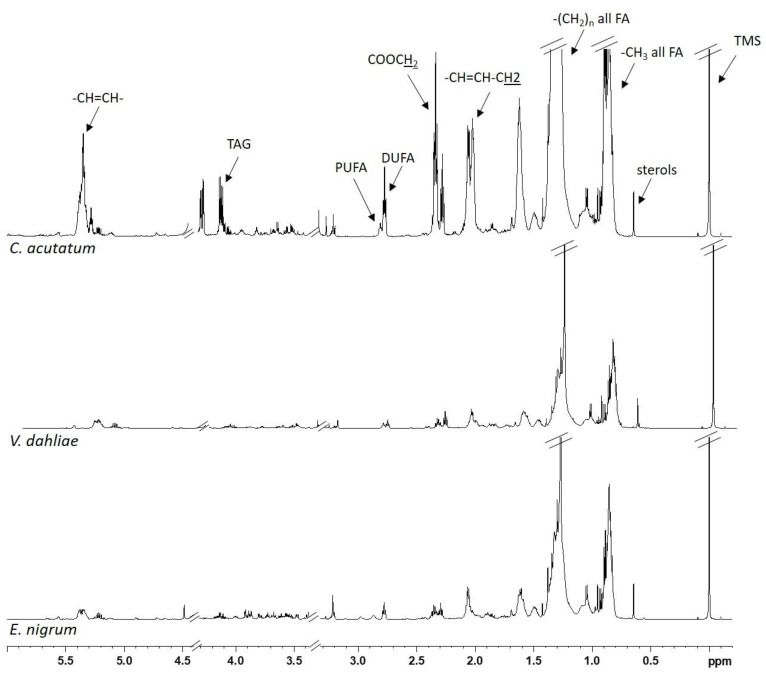
Typical ^1^H NMR spectra of lipid extracts of three pathogens: *E. nigrum* (**bottom**), *V. dahliae* (**middle**) and *C. acutatum* (**top**).

Representative 1D ^1^H NMR spectra of lipid extracts are shown in Figure 4, showing the characteristic signals of fatty acids (saturated, SFAs, mono--, MUFAs, di-, DUFAs, and polyunsaturated, PUFAs, fatty acids), triglycerides (TAG) and phospholipids, in particular phosphatidylcholine (PC) (Table 4).

### 3.4. Multivariate Statistical Analysis

#### 3.4.1. Aqueous Extracts

An exploratory principal component analysis (PCA) of the ^1^H NMR spectra from all aqueous extracts was performed, in order to reveal the main trends of samples. The preliminary unsupervised PCA model was obtained, together with the corresponding t[1]/t[2] score plot, with five principal components being enough to describe more than 80% of the total variance of the entire dataset (t[1] and t[2] explained 38% and 18%, respectively), and R^2^X = 0.843 and Q^2^ = 0.691. The PCA score plot displayed a certain degree of separation among samples (Figure S1). In particular, visual inspection of the PCA showed that *V. dahliae* (obtained from mono- *V. dahliae,* and dual- *V. dahliae* + *E. nigrum*) and *C. acutatum* (obtained from mono- *C. acutatum* and dual- *C. acutatum* + *E. nigrum*) grouped according to mycelium, while *E. nigrum* resulted in two clearly separated sub-clusters on the basis of culture assay performed (mono- *E. nigrum*, or dual-culture assay *E. nigrum* + *V. dahliae* and *E. nigrum* + *C. acutatum*) (Figure S1).

**Figure 5:**
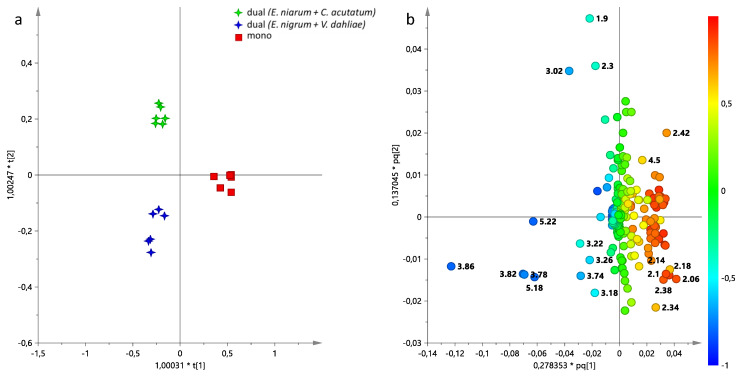
(**a**) OPLS-DA t[1]/t[2] score plot performed on aqueous extracts of *E. nigrum* cultivated in mono- and dual-assay; (**b**) loading plot for the model, colored according to the correlation-scaled vector (p(corr)). The variables indicate the chemical shift value (ppm) in the ^1^H NMR spectra.

In order to better evaluate the differences between the various growth conditions (mono- and dual-culture assays), a supervised analysis was performed for each pathogen. From the preliminary PCA (Figure S1) examination, at first instance, the metabolic profile variations in *E. nigrum* aqueous extracts between mono- and dual-culture assay were assessed. The OPLS-DA analysis for *E. nigrum* gave a good model, with a total variance of R^2^X = 0.918 R^2^Y = 0.959 and predictability of Q^2^ = 0.813 (Figure 5a). In the OPLS-DA score plot, the aqueous extracts of *E. nigrum* in mono-culture assay were well separated along the predictive component t[1] compared to mycelium grown in dual-culture. The molecular components responsible for separation between the different growth conditions were observed by examining the OPLS-DA loading plot for the model (Figure 5b). In particular, higher levels of glutamate, glutamine, succinate and lower levels of sugars (glucose, trehalose, mannitol) were found in *E. nigrum* cultivated in mono-culture assay. Furthermore, *E. nigrum* cultivated in dual-culture assay with *V. dahliae* was characterized from higher levels of ethanolamine, while higher relative content of 4-aminobutyrate and tyrosine were observed in *E. nigrum* cultivated with *C. acutatum*.

**Figure 6:**
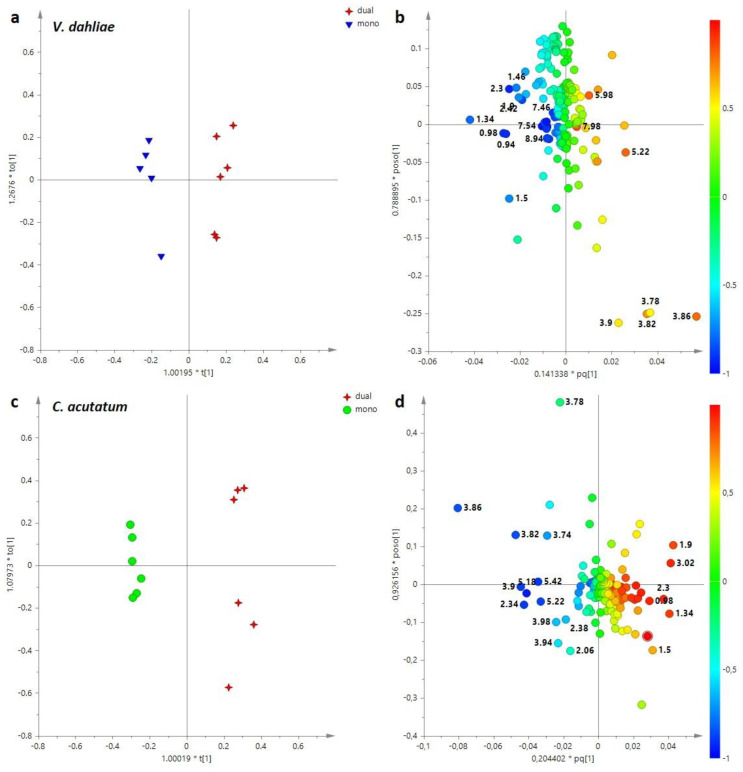
(**a**) OPLS-DA t[1]/to [1] score plot performed on *V. dahliae* aqueous extracts cultivated in mono- and dual-assay; mono, mono-culture assay (*V. dahliae*); dual, dual-culture assay (*V. dahliae + E. nigrum*); (**b**) loading plot for the OPLS-DA model; (**c**) OPLS-DA t[1]/to [1] score plot performed on *C. acutatum* aqueous extracts cultivated in mono- and dual-assay; mono, mono-culture assay (*C. acutatum*); dual, dual-culture assay (*C. acutatum + E. nigrum)*; (**d**) loading plot for the model, colored according to the correlation-scaled vector (p(corr)). The variables indicate the chemical shift value (ppm) in the ^1^H NMR spectra.

The same approach was also used for *V. dahliae* and *C. acutatum*, obtaining two new supervised models (Figure 6). For both mycelia, the OPLS-DA analysis demonstrated a good degree of sample separation based on the different culture conditions.

**Figure 7:**
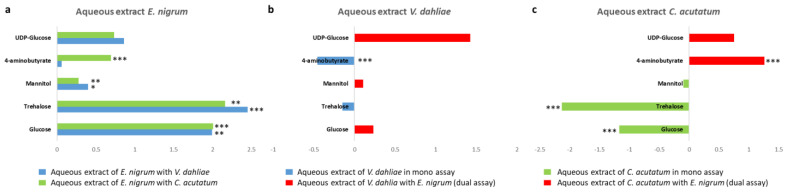
Variation in discriminating metabolite content between mono- and dual-culture assay for aqueous extract; (**a**) *E. nigrum*; (**b**) *V. dahliae*; (**c**) *C. acutatum*. Signif. codes ‘***’ 0.001 ‘**’ 0.01 ‘*’ 0.05.

In particular, the OPLS-DA models obtained with 1 + 2 + 0 components gave, respectively, the following parameters: R^2^X = 0.554, R^2^Y = 0.965, Q^2^ = 0.258 for *V. dahliae* (Figure 6a), and R^2^X = 0.867, R^2^Y = 0.987 and Q^2^ = 0.947 for *C. acutatum* (Figure 7c). Examining the loading plots for the models, it is possible to identify the metabolites responsible for separation. Concerning *V. dahliae*, higher levels of organic acids (lactate, acetate, succinate, 4 aminobutyrate), amino acids (alanine, threonine, valine, leucine, isoleucine, phenylalanine) and nicotinate characterized the mycelium cultivated in mono-assay. Instead, sugars, such as glucose, mannitol and UDP-glucose were found in *V. dahlia* aqueous extracts obtained from co-culture with *E. nigrum* (Figure 6b).

An opposite trend for sugar content was observed for *C. acutatum* compared to *V. dahliae*; in fact, higher levels of glucose, mannitol and trehalose were found in *C. acutatum* cultivated in mono-culture assay (Figure 6d). Moreover, a higher relative content of trigonelline was characteristic of *C. acutatum* obtained from mono-culture. On the other hand, amino acids (in detail: valine, leucine, isoleucine, threonine, tyrosine, phenylalanine), 4-aminobutyrate, malonate and ethanolamine were found at higher levels in *C. acutatum* obtained from co-culture with *E. nigrum* (Figure 6d).

Furthermore, the variation in principal discriminating metabolite content, such as sugars and 4-aminobutyrate, for *E. nigrum*, *V. dahliae* and *C. acutatum* between mono- and dual-culture assay was calculated by the integration of the selected and distinctive NMR signals. In particular, for each mycelium, the differences between the two growth conditions (mono and dual) were observed and reported as Log_2_ Fold Change (FC) ratio in Figure 7. In detail, metabolites at negative values were higher in mono- culture assay, while metabolites at positive values were characteristic of dual-culture assay. Especially, statistically significant differences were observed between *E. nigrum* aqueous extracts obtained from dual-culture assay with *V. dahliae* as well as *C. acutatum* compared to mono-culture assay (Figure 7a).

#### 3.4.2. Lipid Extracts

The same approach applied to aqueous extracts was also performed for lipid extracts, carrying out a preliminary unsupervised analysis of all samples (Figure S2). In the PCA analysis, the total variance of 80% was explained by seven principal components, R^2^X = 0.972 and Q^2^ = 0.920 (with t[1], t[2] of 61% and 17%, respectively). The PCA score plot showed a separation between mono- and dual-culture assay only for *E. nigrum* (mono-, *E. nigrum*, and dual-culture assay *E. nigrum* + *V. dahliae* and *E. nigrum* + *C. acutatum*), while *V. dahliae* (obtained from mono-, *V. dahliae,* and dual-, *V. dahliae* + *E. nigrum*) and *C. acutatum* (obtained from mono-, *C. acutatum* and dual-, *C. acutatum* + *E. nigrum*) samples showed mixed and scattered results.

In order to better understand the differences between *E. nigrum* in mono- and dual-culture assay, analogously to aqueous extracts, the supervised analysis was performed considering *E. nigrum* in different growth conditions, in particular mono-culture assay versus dual-culture assay (*E. nigrum* + *V. dahliae* and *E. nigrum* + *C. acutatum*).

**Figure 8:**
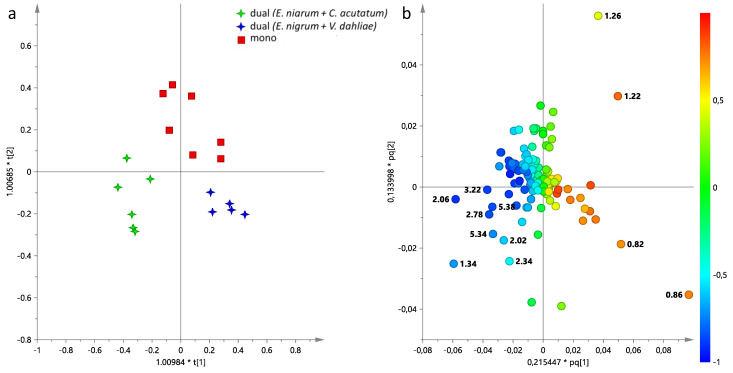
(**a**) OPLS-DA t[1]/t[2] score plot performed on lipid extracts of *E. nigrum* cultivated in mono- and dual-assay; (**b**) loading plot for the model, colored according to the correlation-scaled vector (p(corr)). The variables indicated the chemical shift value (ppm) in the ^1^H NMR spectra.

The supervised OPLS-DA analysis performed on *E. nigrum* gave a good mode (2 + 1 + 0, R^2^X = 0.921 R^2^Y = 0.782 and Q^2^ = 0.655) (Figure 8). In detail, *E. nigrum* samples separated according to culture assay (lipid extracts from mono- and dual-culture assay were found at positive and negative values to the orthogonal component, respectively). Furthermore, OPLS-DA demonstrated a clear separation of *E. nigrum* samples based on co-culture assay along the predictive [1] component (*E. nigrum* + *V. dahliae* at positive values and *E. nigrum* + *C. acutatum* at negative values). From loading plot analysis, it was possible to define the lipids responsible for separation. In particular, *E. nigrum* cultivated in mono-culture was characterized by higher level of SFAs, while higher relative content of phosphatidylcholine (PC), unsaturated fatty acids, principally poly-unsaturated (PUFA) and di-unsaturated (DUFA) were found in *E. nigrum* grown with *C. acutatum*. Instead, *E. nigrum* obtained from co-culture with *V. dahliae* was characterized by greater relative content of short FAs.

**Figure 9:**
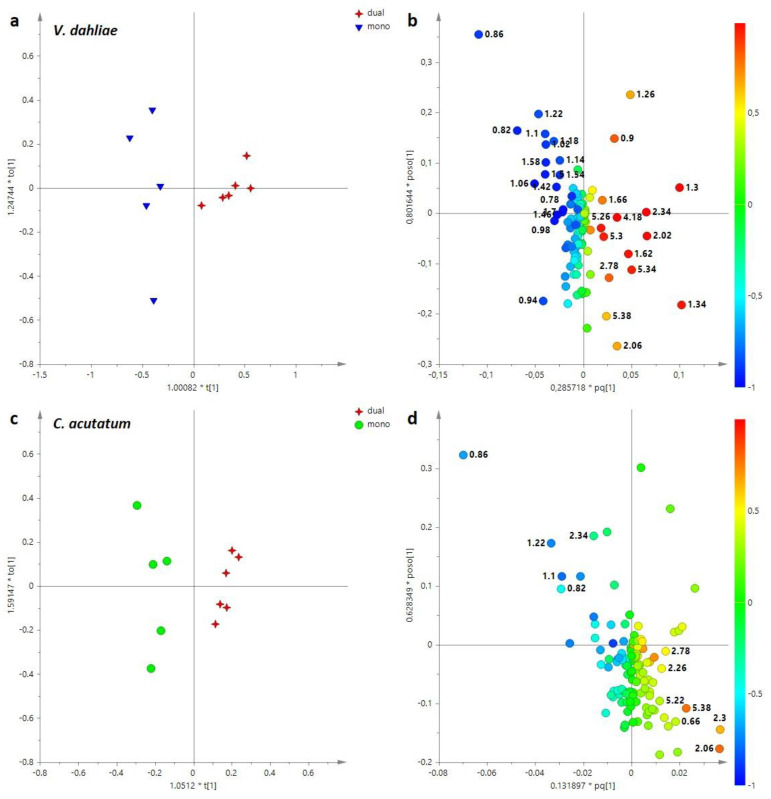
(**a**) OPLS-DA t[1]/to [1] score plot performed on *V. dahliae* lipid extracts cultivated in mono- and dual-assay; mono, mono-culture assay (*V. dahliae*); dual, dual-culture assay (*V. dahliae + E. nigrum*); (**b**) loading plot for the OPLS-DA model; (**c**) OPLS-DA t[1]/to [1] score plot performed on *C. acutatum* lipid extracts cultivated in mono- and dual-assay; mono, mono-culture assay (*C. acutatum*); dual, dual-culture assay (*C. acutatum + E. nigrum)*; (**d**) loading plot for the model, colored according to the correlation-scaled vector (p(corr)). The variables indicate the chemical shift value (ppm) in the ^1^H NMR spectra.

In addition, the differences for *V. dahliae* and *C. acutatum* lipid extracts, between mono- and dual-culture assays respectively, were evaluated by supervised OPLS-DA analysis (Figure 9). Both pathogens showed separation along the predictive component in their respective score plots (Figure 9a,c). The analysis of loading plot (Figure 9b,d) showed for *V. dahliae* as well as for *C. acutatum* higher relative content in short FAs in pathogen lipid extracts obtained from mono-culture assay. By contrast, both pathogens *V. dahliae* and *C. acutatum* obtained from co-culture with *E. nigrum* were characterized by higher relative content of UFAs, such as PUFAs (Figure 9b,d). Furthermore, a greater level of sterol was found in *C. acutatum* grown in dual-culture assay (Figure 9c).

**Figure 10:**
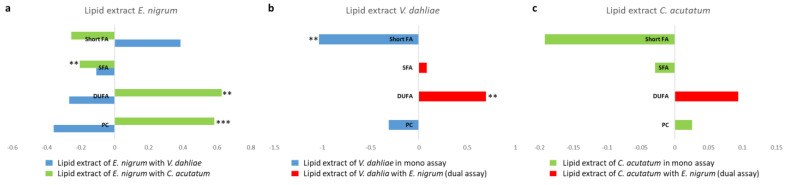
Variation in discriminating fatty acid contents between mono- and dual-culture assay for aqueous extracts; (**a**) *E. nigrum*; (**b**) *V. dahliae*; (**c**) *C. acutatum*. Signif. codes ‘***’ 0.001 ‘**’ 0.01.

In addition, the variations in discriminating fatty acids between different culture assays (mono- and dual-) for each pathogen *E. nigrum*, *V. dahliae* and *C. acutatum* were verified by the integration of unbiased signals (NMR-specific resonances) and reported as Log_2_FC (Figure 10). In bar graphs, fatty acids found at negative values were more prevalent in mono-assay, whereas lipids at positive values characterized the mycelium extract obtained from dual-culture assay. Concerning *E. nigrum* extracts, statistically significant levels of SFA, DUFA and PC were observed principally between mono-culture assay and dual-culture with *C. acutatum* (Figure 10a). No significant differences were observed between mono- and dual-culture assays of lipid *C. acutatum* extracts (Figure 10c).

## 4. Discussion

The olive tree is highly vulnerable to pests and diseases. Those related to fungal infections are highly relevant, although they have not been fully investigated in terms of their specific primary metabolism. The present study investigates the antagonistic potential of *Epicoccum nigrum* (RD4C isolate) against two significant plant pathogens, *Colletotrichum acutatum* (C9D2C isolate) and *Verticillium dahliae* (ER 1357 isolate). Through a combination of NMR-based metabolomic analysis and MVA, we explored the interactions between these fungi and elucidated the biochemical basis of *E. nigrum*’s antagonistic effects. This study demonstrates that *E. nigrum* effectively inhibits the growth of both *C. acutatum* and *V. dahliae*.

The dual-culture assays confirmed the potential of *E. nigrum* as a BCA, with a clear inhibition of both pathogens. Specifically, *E. nigrum* inhibited the growth of *C. acutatum* by 44.97% and *V. dahliae* by 38.73%. These results are consistent with previous studies highlighting the biocontrol potential of *E. nigrum* against various plant pathogens [31,62,63,64]. The observed inhibition percentages indicate a significant antagonistic effect, suggesting that *E. nigrum* could be an effective biocontrol agent for managing these pathogens in agricultural settings.

The NMR-based metabolomics analysis provided detailed insight into the metabolic changes associated with the fungal interactions. Multivariate statistical analysis, mainly OPLS-DA, demonstrated clear metabolic distinctions between mono- and dual-culture assay, for both extracts, aqueous and lipid. In particular, alterations in fungal primary metabolism with changes in sugar, amino acid, organic acid and fatty acid relative contents were observed. It is well known that in the presence of stress, biotic or abiotic, an organism triggers a series of reactions in its metabolism as a defense mechanism to counteract the deleterious effects being caused. Despite the fact that the primary metabolism of fungi plays a crucial role in their response to stresses, pathogenesis and survival, it has been poorly studied so far [56]. On the contrary, considering the potential uses of secondary metabolites in several sectors, such as the pharmaceutical and agri-food industries, the secondary metabolism of pathogenic fungi has been amply studied [56]. Nevertheless, more research is still needed in order to better understand the overall metabolism of these microorganism, since several genes are not expressed under the usual laboratory conditions [56].

In this study, the ^1^H NMR characterization of aqueous and lipid extracts of *E. nigrum*, *V. dahliae* and *C. acutatum* provided advanced information on the metabolite composition of these pathogens. In particular, aqueous extracts revealed a dominance of amino acids, organic acids and sugars, with specific metabolites like propanol in *E. nigrum* and nicotinate in *V. dahliae* being distinctive markers. From statistical analysis, *E. nigrum* showed higher levels of glutamate, glutamine, and succinate in mono-cultures, whereas dual-cultures showed increased ethanolamine and 4-aminobutyrate, indicating a shift in metabolic pathways when in the presence of the pathogens. Similarly, *V. dahliae* and *C. acutatum* exhibited significant metabolic changes in dual-cultures, with higher levels of sugars in *V. dahliae* and increased amino acids and organic acids in *C. acutatum*. These metabolic shifts likely reflect the competitive interactions and stress responses induced by the presence of *E. nigrum*. Among these discriminant metabolites, some are involved in energy and amino acid metabolism and oxidative stress. As is known, in all organisms UDP-glucose is important for several processes in cell physiology [65]. In particular, in fungi, UDP-glucose is implicated in the synthesis of trehalose (osmoprotector) and of β-glucans (essential components of cell walls) [65,66]. Trehalose, a non-reducing disaccharide, is important not only as a reserve carbohydrate, but it also plays a crucial rule in different processes, such as glycolysis control, pathogenicity, sporulation and protection against osmotic stress. Changes in trehalose contents suggest a mechanism of adaptation of plant pathogens to unfavorable or stressful growth conditions [55,56,67]. Additionally, mannitol also showed a shift in relative content between mono- and dual-culture assays. In fungi, mannitol is implicated in several physiological functions, such as carbon storage and pathogenesis [57,68].

In metabolic profiles obtained from aqueous extracts of *E. nigrum*, *V. dahliae* and *C. acutatum* in mono- and dual-assay, variations in amino acid content were also observed. As already reported in the literature, aromatic amino acids (tyrosine and phenylalanine), branched-chain amino acids (leucine, valine, isoleucine) are strictly necessary as precursors in protein synthesis and energy production [65]. Glutamate appears to play a protective role against osmotic stress and acids [56]. Moreover, threonine is important as a nitrogen source for microorganisms, whereas glutamine content reflects the nitrogen state for fungi [56]. In addition, aromatic amino acids represent the essential precursors of numerous secondary metabolites (such as phenylpropanoid, alkaloids) that are produced through several biosynthetic pathways [10]. It is well-known that secondary metabolites are involved in the defense mechanisms of organisms. Furthermore, most fungi convert phenylalanine to phenyl-acetate [69,70]. This latter compound is a bioactive metabolite that is studied not only for its phytotoxicity but also for its antimicrobial activity [70]. In fact, some phenylalanine derivatives have been shown to be effective against both Gram-positive and Gram-negative bacteria, likely due to their interaction with bacterial membranes [71]. However, to our knowledge, there is no evidence in the literature that *Epicoccum* spp. produce phenylalanine-derived antimicrobial compounds.

Furthermore, succinate, 4-aminobutyrate (or GABA) and malonate proved to be discriminating metabolites. In particular, succinate is the main intermediate of the TCA cycle, while the rule of 4-aminobutyrate and its metabolism in phytopathogenic fungi is still unclear, although it is known that 4-aminobutyrate represents a nitrogen and carbon source [72]. Anyway, recent studies have demonstrated that 4-aminobutyrate plays a crucial role in response to environmental stress conditions, with a positive effect on asexual development [72]. In fact, in most fungi, the 4-aminobutyrate is synthesized by decarboxylation of glutamate and it is involved in sporulation [73]. Specifically, in *Alternaria alternata*, GABA metabolism was linked to oxidative stress responses and fungal development, suggesting its potential involvement in pathogenicity and environmental adaptation [74]. Nevertheless, there is no current evidence that *Epicoccum* spp. actively synthesize or utilize GABA as part of their metabolic strategy. Finally, malonate is related to nitrogen metabolism and could play a protective role, also participating in the fatty acid biosynthetic pathways [57].

In this study, besides the aqueous extracts, the lipid profile of *E. nigrum*, *V. dahliae* and *C. acutatum* were further analyzed. The visual inspection of ^1^H NMR spectra revealed the presence of different classes of lipids such as Saturated and Unsaturated Fatty Acids (SFA and UFA, respectively), triglycerides and phospholipids (principally PC). As already observed for aqueous extracts, the OPLS-DA analysis of lipid extracts also exhibited a clear separation between mono- and dual-culture assay for all pathogenic mycelia, buttressing the observed shift in metabolic pathways in the presence of the pathogens. In fungi, lipids represent the major constituent of the membrane system but they occur also as minor components in the cell wall. Lipids can store energy in lipid bodies and, sometimes, lipids act as intra or extracellular signals [75,76]. The variation in lipid composition reflects the greater complexity and cell-size of fungi [76]. The content and the different classes of lipids, in fungi, derive not only from microorganisms but also from developmental stages, nutrition and growth conditions [76]. Furthermore, recent studies suggest a role for lipids in cell signaling, since during host–pathogen interaction there is an exchange of lipid signals between the organisms [75]. Fatty Acids (FA) represent the crucial lipid, being an important energy source and they are incorporated in phospholipids, an essential building block of membranes. Moreover, FAs can be an energy reservoir in the form of TAGs or esters accumulated in lipid drops or globules in cells [75]. As already reported in the literature, in vitro, UFAs (such as linoleic and linolenic acids) are able to reduce the fungal growth in different mycelia [75,77]. TAGs are usually considered as storage lipids, but during mycelium development, they are used as carbon skeletons and energy sources [76]. Besides TAGs, other important lipids are sterols and phospholipids. Sterols influence permeability, thus regulating molecular movement and viscosity of the lipid layer. Furthermore, they also act as precursors of steroid hormones essential in fungal reproduction [76]. In eukaryotes, including most species of fungi, phosphatidylcholine (PC) together with phosphatidylethanolamine are the main phospholipids. Phospholipids are the major constituents of membranes, contributing to their integrity and function, but they also play a crucial rule in fungal vegetative growth [78].

In conclusion, the distinct metabolic profiles observed for both extracts, across mono- and dual-cultures, suggest that the presence of *E. nigrum* may exert its antagonistic effects. This is done through multiple mechanisms, including competition for nutrients, production of inhibitory compounds, and alteration of the pathogens’ metabolic pathways. The changes in relative content of certain amino acids, sugars, organic acids and lipids in the presence of *E. nigrum* indicate a possible disruption of the mycelial metabolic balance, which could hinder its growth and virulence.

## 5. Conclusions

This study provides a comprehensive analysis of the antagonistic interactions in vitro between *E. nigrum* and two major plant pathogens, *V. dahlia* and *C. acutatum*, supported by robust molecular and metabolomic data. The findings highlight the potential of *E. nigrum* as a biocontrol agent and pave the way for further research into its application in agricultural practices. Future studies should focus on field trials to evaluate the practical effectiveness of *E. nigrum* in diverse crop systems and explore the molecular mechanisms underlying its biocontrol activity in greater detail.

By enhancing our understanding of fungal antagonism and metabolic interactions, we can develop more sustainable and effective strategies for managing plant diseases and improving crop health.

## Figures and Tables

**Table 1:** The reference isolates of *Epicoccum* spp. and *Verticillium* spp. used to construct the multilocus phylogenetic tree.

Species	Strain Identification	GenBank Accession Number		References
		ITS	Tub2	
*Epicoccum nigrum*	CBS 140523	MN972952	MN983966	[51]
*E. nigrum*	CBS 125.82	FJ426995	FJ427106	[51]
*E. poae*	LC:8160	KY742113	KY742355	[51]
*E. poae*	LC:8162	KY742115	KY742357	[51]
*E. layuense*	E22	MH643920	MH643930	[51]
*E. layuense*	E23	MH643921	MH643931	[51]
*E. mackenziei*	MFLUCC16-0335	KX698039	MW186824	[51]
*E. dendrobii*	SMEL1	MT020087	MT024597	[51]
*Gibellulopsis* *nigrescens*	PD595	JN187976	-	[48]
*Verticillium albo-atrum*	PD670	JN187990	-	[48]
*V. albo-atrum*	PD748	JN188017	-	[48]
*V. dahliae*	PD332	HQ206718	-	[52]
*V. dahliae*	SDV1025	KC834733	-	[48]
*V. dahliae*	CBS 381.66	KT362918	-	[53]
*V. longisporum*	PD687	HQ206893	-	[48]
*V. longisporum*	PD730	HQ206920	-	[48]
*V. nubilum*	IMI278734	AY935948	-	[48]

**Table 2:** Morphological characteristics of *Verticillium dahliae* and *Epicoccum nigrum* observed on PDA.

Fungal Species	Colony	Conidia
*Verticillium dahliae* (ER 1357)	Initially creamy white, it becomes dark, compact and appressed with age	Hyaline, cylindrical to oval, average length 4.4 ± 1.23 µm (max 6.0 µm)
*Epicoccum nigrum* (RD4C)	Initially white with cottony mycelium, it turns reddish-orange after 14 days; the reverse of the colony produces orange pigments	Globose to subglobose or pyriform, some multicellular; size: 14.5–26.4 µm in length and 15.9–28.4 µm in width

**Table 3:** Chemical shift and assignments of main peaks in the ^1^H NMR spectra of D_2_O extracts of *E. nigrum*, *V. dahliae* and *C. acutatum.* (d: doublet; m: multiplet; s: singlet; t: triplet; q: quartet).

Compound	Assignment	^1^H (ppm, Multiplicity)
Leucine	CH_3_, CH	0.96 (d), 1.70 (m)
Isoleucine	CH_3_, CH	0.90 (t), 1.01 (d)
Valine	CH_3_,CH	0.98 (d), 1.05 (d)
Lactate	CH_3_,CH	1.33 (d), 4.16 (q)
Threonine	γ CH_3_, αCH	1.33 (d), 4.25
Alanine	CH_3_,CH	1.48 (d), 3.79 (m)
Acetate	CH_3_	1.92 (s)
4 amino-butyrate		1.9, 2.29 (t), 3.01
Glutamate	γ CH_2,_ βCH_2_	2.07 (m), 2.36 (m)
Glutamine	γ CH_2,_ βCH_2_	2.14 (m), 2.46 (m)
Succinate	CH_2_/CH_2_	2.41 (s)
Malonate	CH_2_	3.12 (s)
Ethanolamine		3.14
Choline	N(CH_3_)_3_	3.20 (s)
Phosphocholine	N(CH_3_)_3_	3.22 (s)
Mannitol	CH-1	3.68, 3.76, 3.80, 3.86
Trehalose	CH	5.20 (d)
β-Glucose	CH	4.66 (d)
α-glucose	CH-2/CH-6; CH-3/CH5	5.24 (d)
Tyrosine	CH-2/CH-6; CH-4; CH-3/CH5	6.9 (m), 7.20 (m)
Phenylalanine		7.3 (m), 7.38 (m), 7.42 (m)
Adenosine	CH-7; CH-4	8.2, 8.3
Tryptophan		7.53 (d), 7.72 (d)
UDP glucose		7.9, 5.95
Fumarate	CH	6.54 (s)
Formate	CH	8.46 (s)
Nicotinate		8.9, 8.6, 8.25, 7.5
Trigonelline		9.10, 8.84, 8.08

**Table 4:** Chemical shift and assignments of main peaks in the ^1^H NMR spectra of CDCl_3_/CD_3_OD extracts of *E. nigrum*, *V. dahliae*, *C. acutatum*.

Compound	Assignment	^1^H ppm
Sterol		0.63
Short chain length FA	CH_3_	0.82–0.86
FA except ω-3	CH_3_	0.88
All FA	-CH_2_- =CH-CH COOCH_2_CH_2_ COOCH_2_	1.26 1.31 1.55–1.66 2.32–2.37
UFA	CH_2_CH=CH -CH=CH-	1.99–2.10 5.30–5.42
DUFA ω-6	CH=CH-CH_2_-CH=CH	2.78
PUFA	CH=CH-CH_2_-CH=CH	2.81
Phosphatidylcholine (PC)	-N(CH_3_)_3_	3.22
TAG	-CH_2_-	4.17 4.31 5.26
DAG (sn1,2) DAG (sn1,2; sn2,3)	-CH_2_- HO–CH_2_–CH–	4.18 4.35 3.68 5.10

## Data Availability

The data presented in this study are available on request from the corresponding author due to privacy.

## Supplementary Materials

The following supporting information can be downloaded at: https://www.mdpi.com/article/10.3390/jof11020129/s1, Figure S1: PCA score plot t[1]/t[2] (five PCs R^2^X = 0.843 and Q^2^ = 0.691) performed on aqueous extracts of *E. nigrum*, *V. dhaliae* and *C. acutatum*. Abbreviation: mono, mono culture assay; dual, dual culture assay.; Figure S2: PCA score plot t[1]/t[2] (seven PCs R^2^X = 0.972 and Q^2^ = 0.920) performed on lipid extracts of *E. nigrum*, *V. dhaliae* and *C. acutatum*. Abbreviation: mono, mono culture assay; dual, dual culture assay.
