# Supplementary material for: Evaluation of the Effects of Epicoccum nigrum on the Olive Fungal Pathogens Verticillium dahliae and Colletotrichum acutatum by 1H NMR-Based Metabolic Profiling
Source: J Fungi (Basel). 2025 Feb 8;11(2):129. doi: 10.3390/jof11020129 (PMC11856019; doi:10.3390/jof11020129)
Supplement: Supplementary file 1 [file jof-11-00129-s001.zip › jof-3450144-supplementary.pdf]

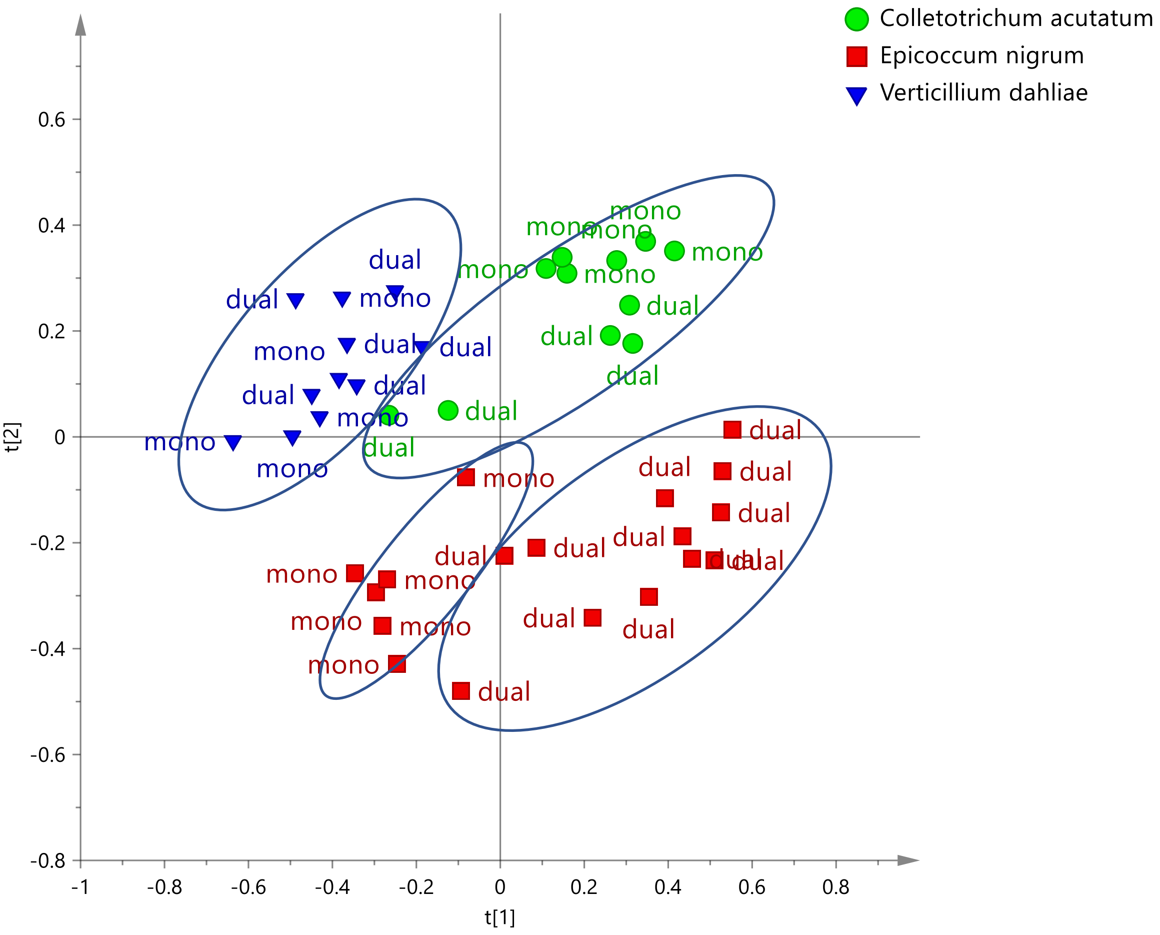

Figure S1. PCA score plot t[1]/t[2] (five PCs R^2X= 0.843 and Q^2= 0.691) performed on aqueous extracts of *E. nigrum*, *V. dhaliae* and *C. acutatum*. Abbreviation: mono, mono culture assay; dual, dual culture assay.

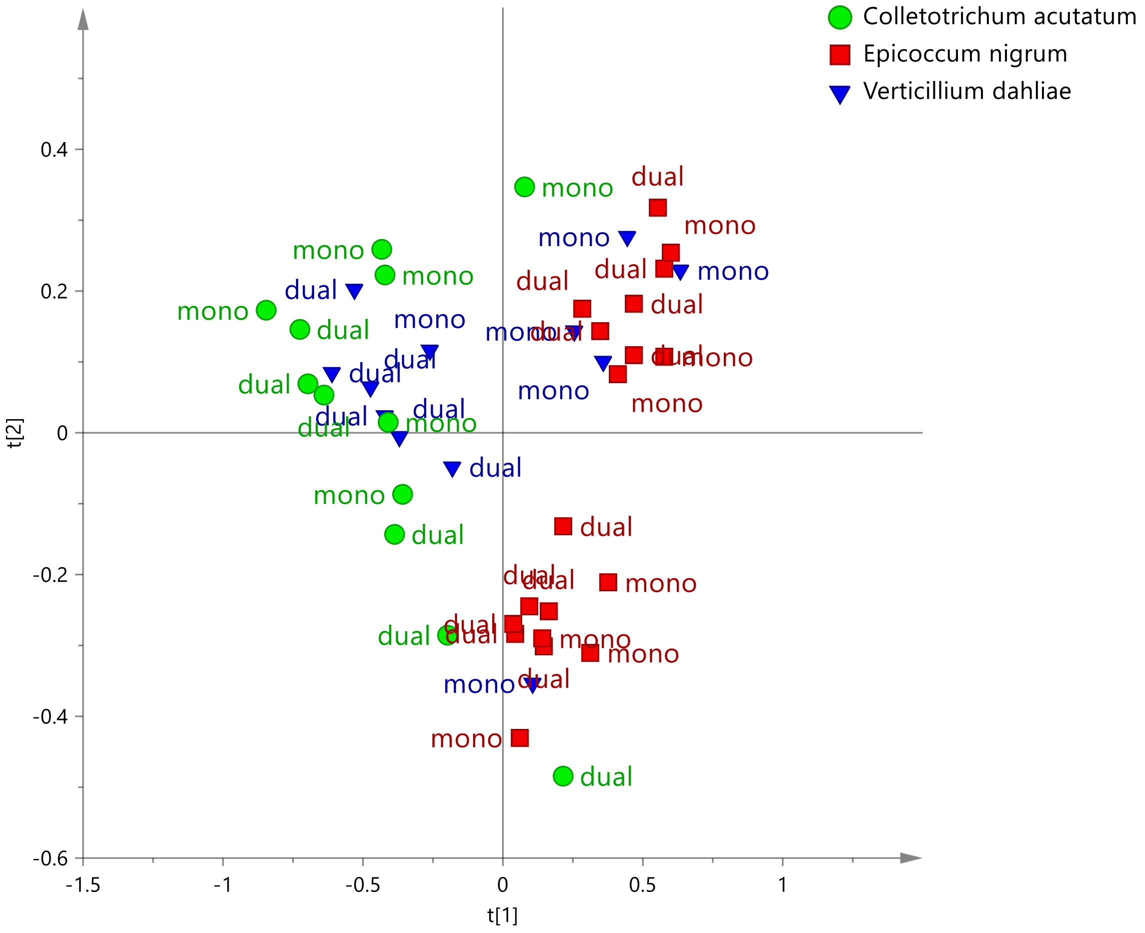

Figure S2. PCA score plot t[1]/t[2] (seven PCs $R^2X= 0.972$ and $Q^2= 0.920$) performed on lipid extracts of *E. nigrum*, *V. dhaliae* and *C. acutatum*. Abbreviation: mono, mono culture assay; dual, dual culture assay.
